# Comparative Genomics Analysis of *Lactobacillus mucosae* from Different Niches

**DOI:** 10.3390/genes11010095

**Published:** 2020-01-14

**Authors:** Yan Jia, Bo Yang, Paul Ross, Catherine Stanton, Hao Zhang, Jianxin Zhao, Wei Chen

**Affiliations:** 1 State Key Laboratory of Food Science and Technology, Jiangnan University, Wuxi 214122, China; jymelody1118@163.com (Y.J.); zhanghao61@jiangnan.edu.cn (H.Z.); zhaojianxin@jiangnan.edu.cn (J.Z.); chenwei66@jiangnan.edu.cn (W.C.); 2 School of Food Science and Technology, Jiangnan University, Wuxi 214122, China; 3 International Joint Research Center for Probiotics & Gut Health, Jiangnan University, Wuxi 214122, China; p.ross@ucc.ie (P.R.); catherine.stanton@teagasc.ie (C.S.); 4 APC Microbiome Ireland, University College Cork, T12 K8AF Cork, Ireland; 5 Teagasc Food Research Centre, Moorepark, Fermoy, P61 C996 Cork, Ireland; 6 National Engineering Research Center for Functional Food, Jiangnan University, Wuxi 214122, China; 7 Wuxi Translational Medicine Research Center and Jiangsu Translational Medicine Research Institute Wuxi Branch, Wuxi 214122, China; 8 Beijing Innovation Center of Food Nutrition and Human Health, Beijing Technology and Business University (BTBU), Beijing 102488, China

**Keywords:** *Lactobacillus mucosae*, comparative genomics, enterolysin A, carbohydrate, CRISPR-Cas, EPS

## Abstract

The potential probiotic benefits of *Lactobacillus mucosae* have received increasing attention. To investigate the genetic diversity of *L. mucosae*, comparative genomic analyses of 93 strains isolated from different niches (human and animal gut, human vagina, etc.) and eight strains of published genomes were conducted. The results showed that the core genome of *L. mucosae* mainly encoded translation and transcription, amino acid biosynthesis, sugar metabolism, and defense function while the pan-genomic curve tended to be close. The genetic diversity of *L. mucosae* mainly reflected in carbohydrate metabolism and immune/competitive-related factors, such as exopolysaccharide (EPS), enterolysin A, and clustered regularly interspaced short palindromic repeats (CRISPR)-Cas. It was worth noting that this research firstly predicted the complete EPS operon shared among *L. mucosae*. Additionally, the type IIIA CRISPR-Cas system was discovered in *L. mucosae* for the first time. This work provided new ideas for the study of this species.

## 1. Introduction

*Lactobacillus mucosae*, as a potential probiotic, has attracted much attention. In industrial application, *L. mucosae* can produce not only propionic acid to improve the wet fermentation of beer, grain, and rumen [1] but could also generate exopolysaccharide (EPS) to serve as a thickener and stabilizer for yogurt [2] and cheese [3]. In addition, EPS also has associated healthy benefits, such as anti-inflammation, reducing blood fat, and lowering cholesterol [4], which provides a basis for the related animal model. It has been reported that *L. mucosae* has antitoxin and antibacterial activity, which could clear up the Zen toxin [5], and inhibit a variety of Gram-positive and negative pathogens [6], such as *Escherichia coli*, *Salmonella typhimurium* [7], and *Staphylococcus* [8].

*L. mucosae* was originally found in the small intestine of piglets [9], and often occurred in the rumen of cattle later [10]. A strain of *L. mucosae* isolated from goat milk was recently reported [11]. Although *L. mucosae* has been shown to have a number of benefits to human, only two human-derived strains have been reported yet, which were isolated from childhood feces [12,13]. This status quo may limit the excavation of other functions of *L. mucosae*, which requires more strains. With the rapid development of bioinformatics, comparative genomics provides a novel way to effectively assess the genetic diversity of bacteria [14], which could explore the origin of the strain and determine the genetic distribution of a particular species [15]. In 2017, Valeriano et al. [6] firstly analyzed the potential specific genes and intestinal adaptability of *L. mucosae* LM1 through genome comparison, such as the average nucleotide identity (ANI) analysis with other species and collinearity analysis within species; unfortunately, the number of strains involved was limited. Heretofore, the comparative genomic research on *L. mucosae*, particularly the genes involved in carbohydrate utilization, bacteriocin, and the CRISPR system, has not been conducted. In the current work, to explore the genetic diversity and potential host adaptation of *L. mucosae*, genome sequencing and comparative genomics analysis were performed for strains isolated from different niches.

## 2. Materials and Methods

### 2.1. Identification and General Genome Features

In total, 93 *L. mucosae* strains were isolated from different samples from different niches and regions among China previously in our lab. Genomic sketches of all those *L. mucosae* strains were sequenced by an Illumina Hiseq × 10 platform (Illumina, San Diego, CA, USA) to generate a 2 × 150 bp paired-end library and construct a paired-end library using an average read length. It used double-ended sequencing with a single-ended sequencing reading of 150 bp. The reads were assembled by SOAPde-novo and local inner gaps were filled with the software GapCloser [16]. The alignment was performed using eight *L. mucosae* strains of the currently disclosed genome, wherein LM1 isolated from the pig small intestine was used a model strain.

### 2.2. The Average Nucleotide Identity (ANI) Values and Phylogenetic Analyses

ANI analysis was carried out to evaluate the relationship among the species with a threshold greater than 95% being the same species. The method of calculating ANI was based on the average of the identity of the homologous genes of each pair of sequences [6]. The protein sequences extracted from those 101 strains were aligned using Orthomcl software (maintaining 50% identity; the cut-point parameter E-value was 1e–4) [17]. The Markov Cluster Algorithm (MCL) clustering algorithm (expansion index of 2.50) was used to cluster protein families with the same function [18]. After the orthologous genes (OGs) were extracted, Interactive Tree Of Life (iTOL) was performed to construct a phylogenetic tree [19].

### 2.3. Pan-Genome and Core-Genome Analysis

PGAP v1.2.1 was used to calculate the pan-genome and core-genome [20]. The evolutionary genealogy of genes: Non-supervised Orthologous Groups (EggNOG) database was used to classify the function of the core genes [21].

### 2.4. Genotype/Phenotype Association Applied to Carbohydrate Metabolism

Carbohydrate utilization gene annotations were performed by the Carbohydrate Active Enzyme Database (CAZy) [22]. The strains were clustered using HemI software [23].

The phenotype of carbohydrate utilization was tested for each strain and the steps were briefly described as follow. Twenty-four different carbohydrates, including D-galactose, D-lactose, maltose, fucose, D-ribose, D-fructose, L-arabinose, sucrose, D-xylose, raffinose, α-lactose, fructo-oligosaccharide (FOS), Xylo-oligosaccharide (XOS), gum arabic, celliboose, D-mannitol, D-mannose, 2′-fucosyllactose (2′-FL), D-sorbitol, trehalose, rhamnose, esculin, pinotriose, and salicin, were selected for carbohydrate utilization analysis. Then, 10% (*w*/*v*) aqueous solution of these carbohydrates were prepared, filtered using a 0.22-μm sterile membrane filter, and stored at 4 °C prior to use. The assay medium without glucose was freshly prepared while the ratio of the other substances was the same as that of the MRS medium, and bromocresol purple was added to the medium as an indicator. After autoclaving and cooling, the sterile carbohydrate aqueous solution was mixed to the medium at a final concentration of 1%. To test the sugar utilization capacity of each strain, after being sub-cultured twice, the strains were inoculated into test growth medium with a 1% inoculum, and each medium was supplemented with a different sugar. After anaerobic incubation for 12 h at 37 °C [24], the utilization was observed by color. All the test was performed in triplicate.

### 2.5. Prediction of the EPS Gene Operon

The protein sequence of the tested strain was aligned with the protein sequence of the EPS-encoding operon using the Basic Local Alignment Search Tool (BLAST) program [25]. The presence of genes was determined based on the alignment fragment size and identity [26].

### 2.6. Prediction of Bacteriocin Operon

BAGEL3 was performed to mine genomes for potential bacteriocin operons [27]. The domains of bacteriocin were determined using BLASTP analysis against the non-redundant protein databases created by BLASTP based on National Center for Biotechnology Information (NCBI).

### 2.7. CRISPR-Cas Identification and Characterization

The Clustered Regularly Interspaced Short Palindromic Repeats (CRISPR) region and the CRISPR-related protein (Cas) were predicted using the CRISPR CasFinder [28], and neighbor-joining trees based on Cas protein were built [29]. The sequence of conserved direct repeats (DRs) were visualized by WebLogo [30].

## 3. Results

### 3.1. General Genome Features of L. mucosae

Previously in our lab, 93 *L. mucosae* strains were isolated from different samples, including fecal samples of humans at different ages, animal feces, human vaginal tract, and milk, etc. The draft genomes of all those strains were sequenced via the next generation sequencing (NGS) approach. The genome of *L. mucosae* was from 1.86 to 2.45 Mb, with a mean size of 1.79 Mb, and displayed an average G + C content of 48.1%. The number of tRNA genes in most strains was between 40 and 67, whereas one strain had tRNA genes less than 40 and one reached 79 (Table 1).

### 3.2. ANI and Phylogenetic Analyses of L. mucosae

**Figure 1:**
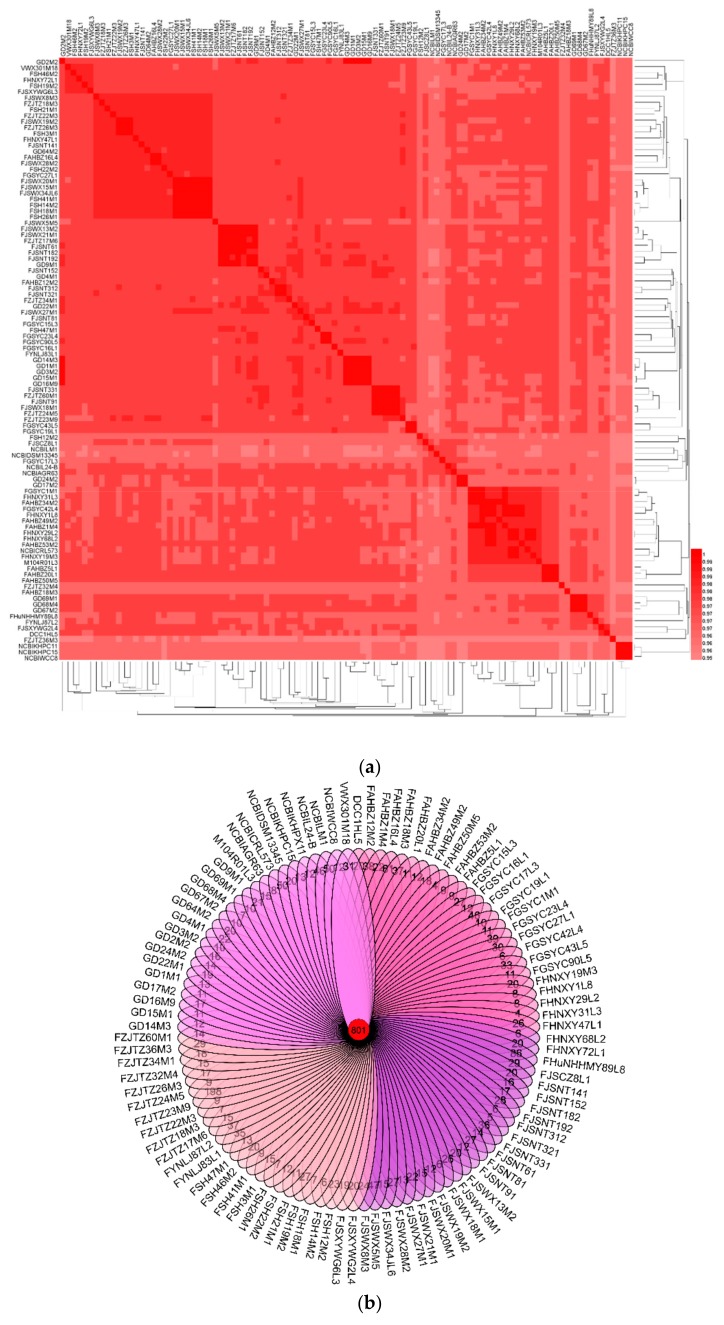

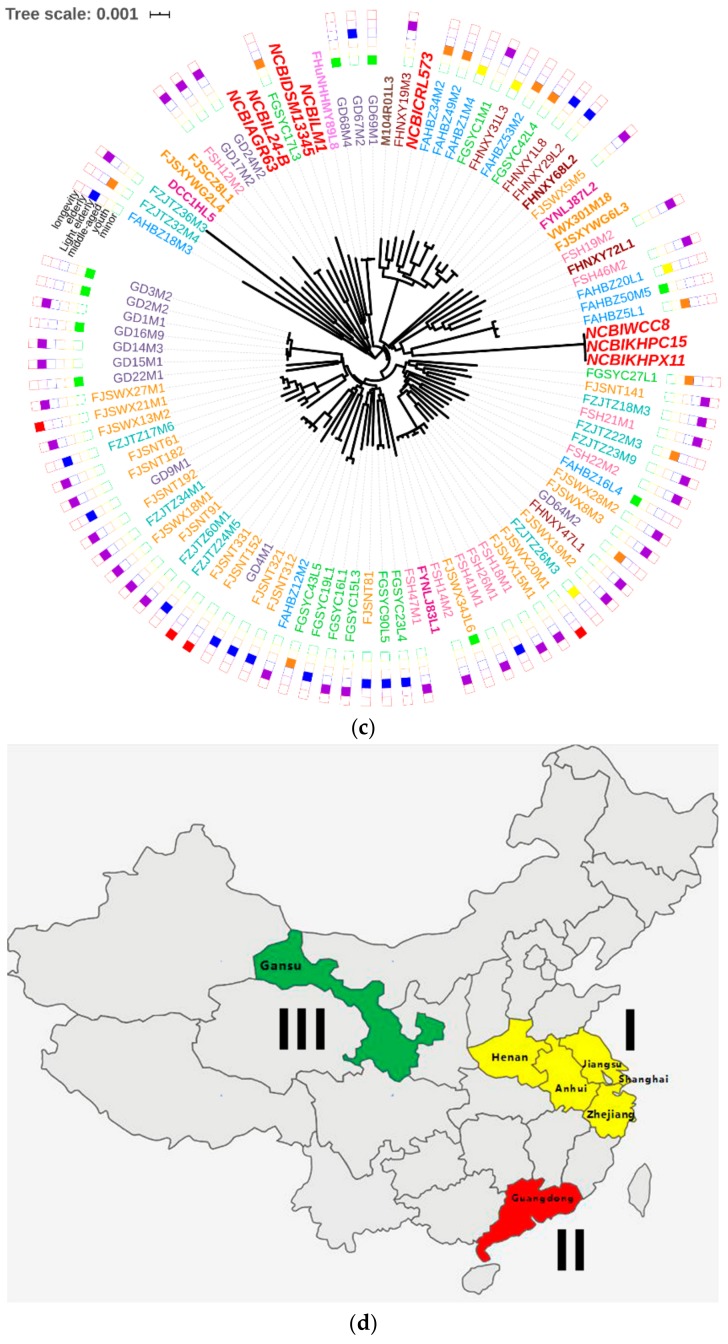
The Average Nucleotide Identity (ANI) and phylogenetic analysis of *L. mucosae*: (**a**) Heatmap showing the ANI value among 101 *L. mucosae* strains. Proposed species cut-off boundary was around 95% to 96%. (**b**) Venn diagram based on homologous genes. (**c**) Phylogenetic tree based on orthologous genes, taking into account factors, such as distance, age, and habitat. Th different colored strain numbers represent the strains isolated from different sampling points. Green, orange, cyan, blue, pink, brown, and purple representing Gansu, Jiangsu, Zhejiang, Anhui, Shanghai, Henan, and Guangdong, respectively. (**d**) Display of the sampling area in the Chinese map. Yellow representing Area I, red representing Area II, and green representing Area III.

To explore the biology of *L. mucosae*, all those 93 newly sequenced genomes combined with eight publicly available *L. mucosae* genomes (LM1, DSM13345, DPC6424, WCC8, KHPC15, KHPX11, AGR63, and L24-B) were loaded to the ANI analysis. The results showed that the ANI valued from 0.953977 to 0.999552 (Figure 1a), which indicated that all of those strains were *L. mucosae* without any potential subspecies.

In order to evaluate the genetic distance among strains, the phylogenetic relationship among all those strains was studied. OGs among all those *L. mucosae* were 801 genes (Figure 1b). The phylogenetic tree was constructed using the protein-coding sequences of those 801 OGs (Figure 1c), which represented the relevance of the strains. The three strains, KHPC15, KHPX11, and WCC8, isolated from the rumen of cattle, were located in the same small branch in the phylogenetic tree. Additionally, similar results were found for LM1 and DSM13345, which were both originally from piglet small intestine and located in the same branch in the tree. However, some strains from different niches shared the same branch, such as FHNXY72L1 (dog derived) and SH46M2 (human derived), FHNXY68L2 (dog derived), and FHNXY29L2 (human derived), respectively.

In terms of human-derived strains, the geographical distance and age of the samples were considered as two potential key factors. Considering the geographical distance, the sampling points were mainly divided into three parts (Figure 1d): Henan, Jiangsu, Zhejiang, Shanghai, and Anhui (Area I); Guangdong (Area II); and Gansu (Area III). The strains isolated from Area II and Area III were concentrated in a large branch, and only a few strains were dispersed. The number of strains isolated from Area I was substantial. Most of the isolates from Jiangsu, Shanghai, and Zhejiang were concentrated (coastal region), and most of the isolates from Anhui and Henan were concentrated in a large branch (inland region). In addition, the strains isolated from humans with different ages were widely distributed, and the strains from the similar age group (minors, young, middle-aged, light-elderly, elderly, and longevity) did not show obvious aggregation. There was no significant correlation between the relationship of the diversity of strain and the age of the host.

### 3.3. Pan-Genome and Core Genes of L. mucosae

**Figure 2:**
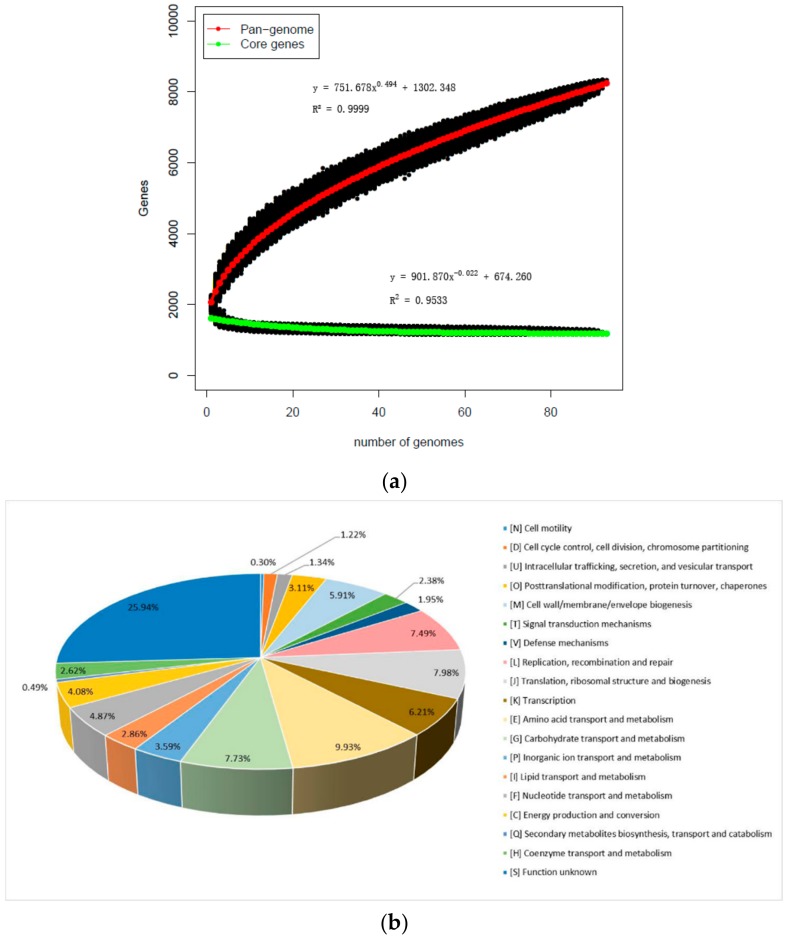
Pan-genome and core genes of *L. mucosae*: (**a**) Numbers of total features in the core (green) and pan (red) genome as a function of the number of strains sequenced. (**b**) Percentage of genes associated with general cluster of orthologous groups (COGs) of proteins’ functional categories.

In order to further study the genetic diversity of *L. mucosae*, the pan-genome and core genes were analyzed. Based on those newly sequenced and eight publicly available genomes of *L. mucosae*, a total of 101 genomes were included. The number of core-genes and pan-genes, with the number of sequenced strains, were used to draw a functional relationship diagram. It showed that the slope of the number of core-genes was close to the asymptote, and even after the 101 genomes had been compiled, while the pan-genomic curve gradually closed. Specifically, in the first two iterations of the pan-genome curve, each genome increased with an average of 302 gene families, and reduced to 35 genes with the average of the last two additions, generating a total of 8753 pan-genes. Consistently, the core genome reached the value of 755 genes in the last iteration (Figure 2a). With functional analysis for the core genes of *L. mucosae*, the core genome included genes for replication, transcription, translation, central and cell wall metabolism, biosynthesis of amino acids and metabolism of nucleotides, fatty acids, and phospholipids. Among them, the genes related to carbohydrate metabolism of *L. mucosae* accounted for ~32.10% of the core genome while the role of ≈25.95% of the core-genome was unknown. Those unknown genes were found to be uncharacteristic as conserved proteins. In addition, 1.94% out of the core genes was involved in the defense function of bacteria (Figure 2b).

### 3.4. In Silico Gene–Trait Matching for Carbohydrate Utilization

**Figure 3:**
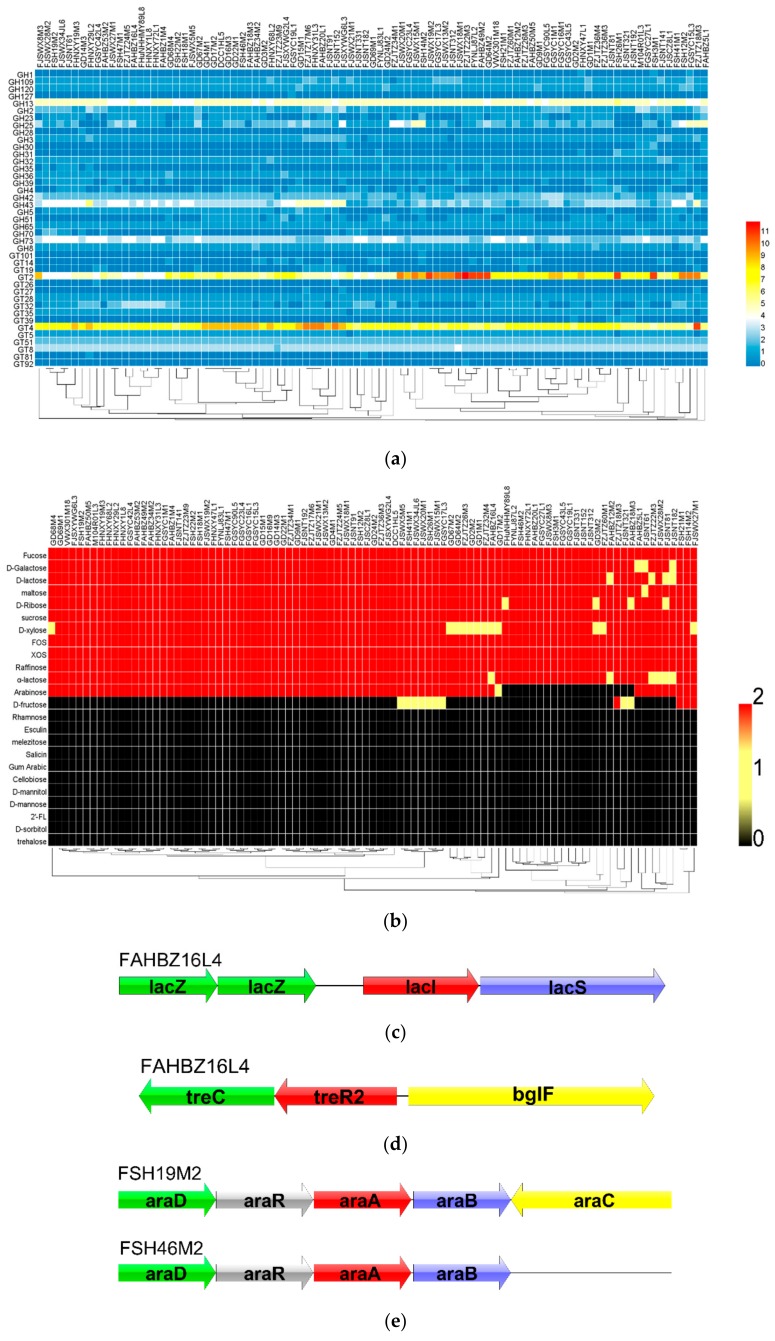

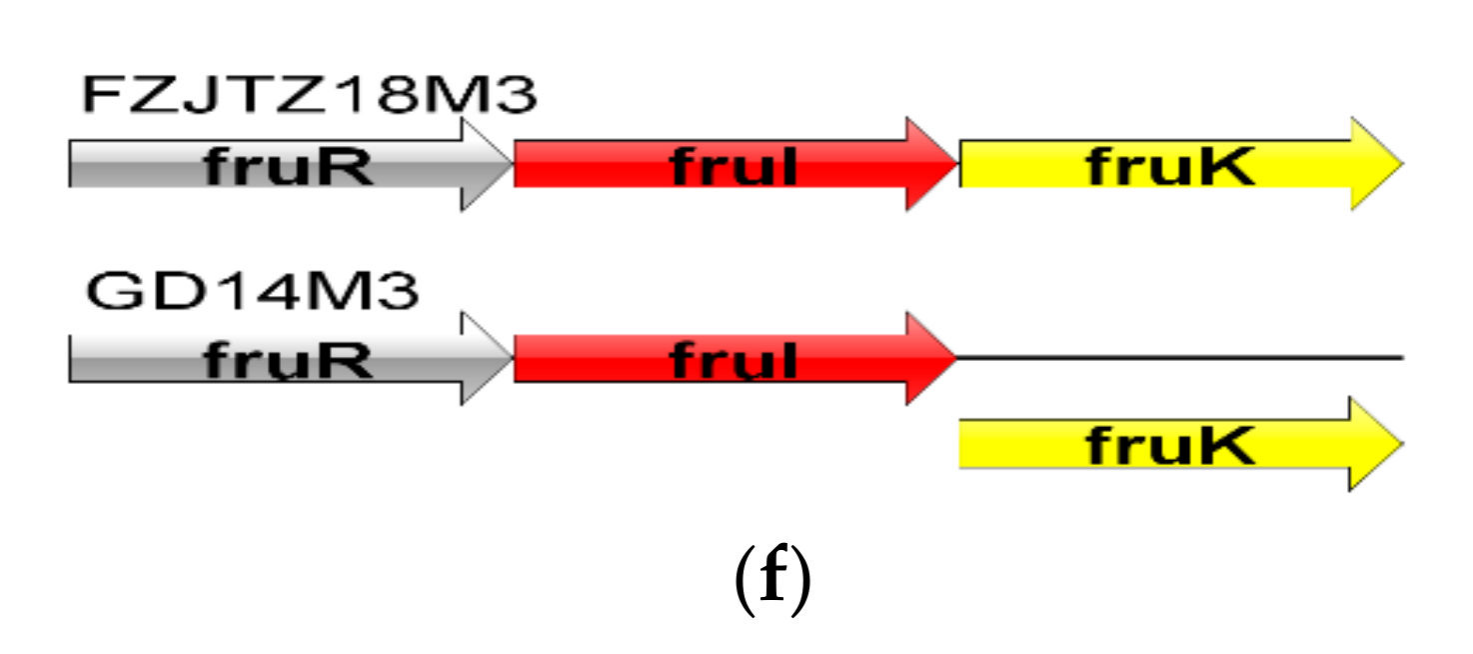
Genotype-phenotype analysis of carbohydrate utilization of 93 *L. mucosae* strains: (**a**) Utilization of 24 kinds of carbohydrates. (**b**) Predicted glycoside hydrolases and transferase\prediction of gene cluster for utilization of lactose (**c**), trehalose (**d**), L-arabinose (**e**), and D-fructose (**f**).

In order to expand the understanding of carbohydrates utilization of *L. mucosae*, the CAZy database was used to analyze all the 93 sequenced genomes. The results revealed that *L. mucosae* contained genes encoding predicted carbohydrate-active enzymes, including 25 glycosyl hydrolase (GH) families and 17 glycosyl transferase (GT) families (Figure 3a). GH2 (β-galactosidase (EC 3.2.1.23)), GH13 (α-glucosidase (EC 3.2.1.20)), GH32 (invertase (EC 3.2.1.26)), GH36 (α-galactosidase (EC 3.2.1.22)), GH42 (β-galactosidase (EC 3.2.1.23)), GH43 (β-xylosidase (EC 3.2.1.37); α-L-arabinofuranosidase (EC 3.2.1.55)), GH65 (maltose phosphorylase (EC 2.4.1.8)), GH73 (lysozyme (EC 3.2.1.17)), GH109 (α-N-acetylgalactosaminease (EC 3.2.1.49)), and GH120 (β-xylosidase (EC 3.2.1.37)) were distributed among all the strains. The remaining 15 GHs have different distributions in those strains. Among them, GH13 and GH43 accounted for a relatively high proportion in the GH family. They were associated with the degradation of alpha-glucopyranose units and long-chain carbohydrates, respectively. Additionally, among all the predicted GT families, GT2 and GT4 were the most abundant, which were mainly involved in the synthesis of EPS. However, according to the clustering results of carbohydrate utilization enzymes, there was no obvious relationship with the sampling region, age, and habitat of the host; meanwhile, some strains had no obvious regularity in the clustering results.

To verify the genotype, the utilization phenotype of *L. mucosae* on 24 sugars as a unique carbon source was tested individually. All the strains were found to be able to grow with glucose, which was used as a positive control in the test. While all the tested strains were able to ferment D-galactose, D-lactose, maltose, fucose, D-ribose, sucrose, D-xylose, raffinose, α-lactose, FOS, and XOS, they were unable to utilize gum arabic, celliboose, D-mannitol, D-mannose, 2′-FL, D-sorbitol, trehalose, rhamnose, esculin, pinotriose, and salicin (Figure 3b). However, the metabolic levels of D-xylose, D-lactose, α-lactose, D-fructose, and L-arabinose were different. Among them, 79.6% strains did not use arabinose completely, and only 13.9% strains could utilize fructose. In addition, with those results, the ability of strains utilizing different carbohydrates were independent of the habitat, geographical distance, and age of the host.

An in silico assessment of the role of specific genes associated with sugar metabolism was performed with the gene–trait matching (GTM) analysis according to the association between the presence or absence of gene families, and growth or non-growth phenotype of the 93 *L. mucosae* strains. The β-galactosidase belonging to the GH2 and GH42 families was responsible for the metabolism of D-galactose, D-lactose, and α-lactose. Although some strains differed in the utilization of each sugar, all the strains containing GH2 and GH42 were able to utilize these three sugars. The genotype and phenotype reached a 100% match. Further analysis of the lactose gene cluster revealed that all the strains contained intact lactose operons, involving *lacS* (PTS sugar transporter subunit IIA), *lacZ* (β-galactosidase), and *lacI* (*LacI* family transcriptional regulator) (Figure 3c).

The key enzyme for hydrolyzing trehalose was alpha-phosphotrehalase, which belonged to the GH65_29 family. Although all the strains contained the GH65 family, the gene *treC* encoding alpha-phosphotrehalase was only found in the strain of FGSYC17L3. By investigating the genome of the strain FGSYC17L3, a complete trehalose operon that presented in *L. mucosae* was found, which was mainly composed of *treC*, *treR2* (trehalose operon repressor), and *bglF* (PTS trehalose transporter subunit IIBC) (Figure 3d). However, the strain FGSYC17L3 did not utilize trehalose, which was inconsistent with the genotype.

In addition, considering the significant difference in the metabolic capacity of L-arabinose and D-fructose, the gene clusters of these two sugars were analyzed. The usage of L-arabinose was mainly related to L-ribose-5-phosphate-4-isomerase (*araD*), L-arabinose isomerase (*araA*), and related transcriptional regulators (Figure 3e). Although all the strains contained *araA*, 19 strains lacked transcriptional regulators, hence, no strains were observed to grow in the medium with arabinose as the sole carbon source. The utilization of D-fructose required fructokinase (*fruK*), phosphoglucose isomerase (*fruI*), and the intact ABC transport system (Figure 3f). Fructokinase phosphorylated intracellular fructose to fructose-6-phosphate, which was isomerized to glucose 6-phosphate by phosphoglucose isomerase. However, those functions were carried out under the premise of the ABC transport system. In total, 86% of the strains in this study could not metabolize fructose due to the absence of the ABC transport system.

### 3.5. Prediction of the EPS Operon in L. mucosae

**Figure 4:**
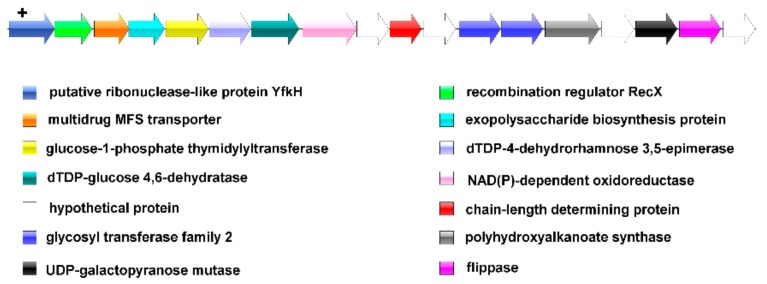
Prediction of exopolysaccharide (EPS) synthesis gene cluster in *L. mucosae.*

EPS production was one of the characteristics of *L. mucosae*. To explore whether the newly genome-sequenced *L. mucosae* could produce EPS, the gene operon was predicted by BlastN. The results showed that 16 out of 93 *L. mucosae* strains consisted of the EPS-producing operons, and all of those were the same type. The EPS gene cluster in *L. mucosae* mainly composed of extracellular polysaccharide biosynthesis protein, chain length-determining protein, *rfbA* (glucose-1-phosphate thymidyltranseferase), *rfbC* (dTDP-4-dehydrorhamnose 3,5-epimerase), *rfbB* (dTDP-glucose 4,6-dehydratase), *rfbD* (NAD(P)-dependent oxidoreductase), glycosyltransferase (GT2), ribonuclease, and flipping enzyme (Figure 4). Except for the individual differences in the third hypothetical proteins, the number and order of key genes for EPS production among 16 strains were matched. Glucose-1-phosphate thymidyltranseferase was responsible for the first step of catalyzing the synthesis of polysaccharides by transferring a sugar-1-phosphate molecule to a lipid carrier located on the cell membrane. Other GT was responsible for catalyzing the synthesis of glycosidic bonds between the new monosaccharide molecule and the sugar molecule on the lipid carrier, thereby forming a repeating unit of the polysaccharide. After the unit structure of the polysaccharide was synthesized, it was exported to the cell surface through a polymer transfer pathway, which was catalyzed by a flippase.

### 3.6. Prediction of Bacteriocin Production in L. mucosae

**Figure 5:**
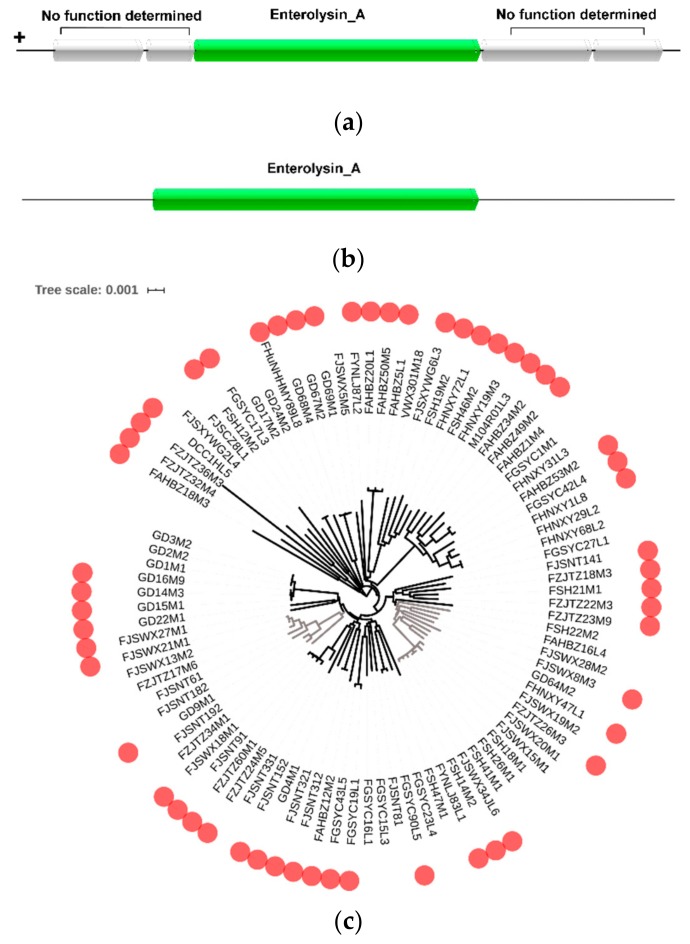
Prediction of bacteriocin operon in *L. mucosae*: (**a**) Predicted gene cluster for enterolysin A synthesis in most strains of *L. mucosae*. (**b**) Predicted gene cluster for enterolysin A synthesis in the strain DCC1HL5. (**c**) Distribution of complete bacteriocin operons.

In order to investigate the potential bacteriocin produced by *L. mucosae*, BAGEL was used to predict the bacteriocin operon. Totally, 77 enterolysin A operons were found in those 93 genomes. Among them, seven strains contained 2–4 enterolysin A operons. The *enlA* gene was a key gene for the synthesis of enterolysin A. Through observation of the strain gene cluster, the upstream and downstream genes of the *enlA* were hypothetical proteins that were not related to the synthesis of enterolysin A (Figure 5a). There was even only a single *enlA* gene in the gene cluster of the strain DCC1HL5 (Figure 5b). It was speculated that the single *enlA* gene can synthesize enterolysin A. In addition, according to the results from BAGEL, the bacterial bacteriocin production was further analyzed based on the phylogenetic tree (Figure 5c). Although the strains encoding enterolysin A had relatively large clusters in phylogenetic trees, such as from FSH22M2 to FSH14M2 and from FJSWX21M1 to FZJTZ34M1, they appeared to be independent on the host.

### 3.7. CRISPR-Cas Systems in L. mucosae

**Figure 6:**
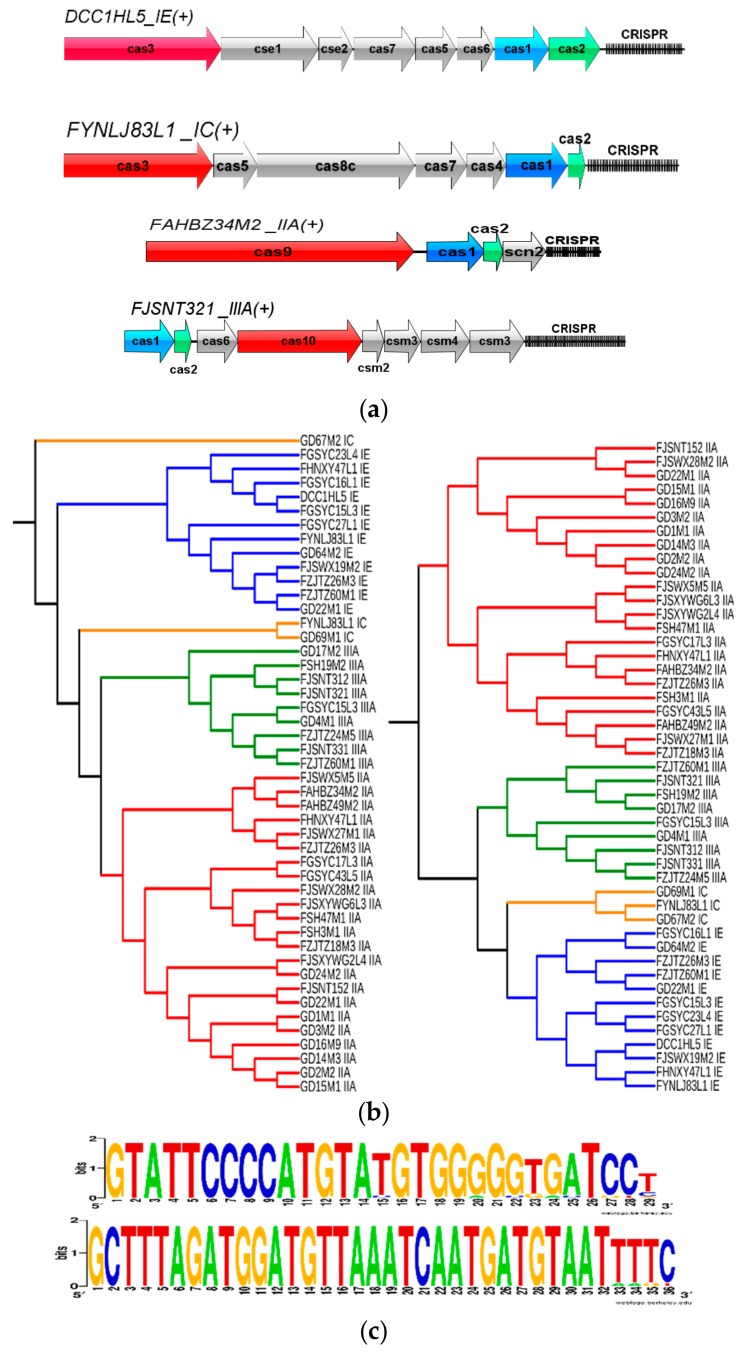
CRISPR-Cas systems in *L. mucosae*: (**a**) CRISPR loci in *L. mucosae* (four subtypes). The CRISPR locus was annotated and depicted with signature *Cas* genes colored in red, Cas3 for Type I, Cas9 for Type II, and Cas10 for Type III, and the universal Cas1 and Cas2 were colored in blue and green, respectively. Accessory genes were colored in a gray scale. CRISPR is represented using the fence graphics on the right side of each locus. (**b**) The phylogenetic tree constructed with Cas1 and DR sequences with four different subtypes in four colors. (**c**) Three kinds of Direct Repeats (DR) sequences in the same strain were visualized by using WebLogo. The height of the letter represented the frequency of the corresponding base at that position.

To explore the acquired immunity, the presence of the CRISPR-Cas system was investigated through in silico analyses. CRISPR was found in all those 93 genomes, and only those with higher levels of evidence were considered in the current study. On the other hand, the CRISPRs without Cas protein were ignored due to the lack of ability to silence foreign DNA. In total, 41 strains carrying 47 total CRISPR-Cas systems were identified. Among those strains, four CRISPR-Cas subtypes were found, including type IE (12 strains), type IC (three strains), type IIA (23 strains), and type IIIA (nine strains). However, the existence and subtypes of the CRISPR-Cas system seemed to have little correlation with the source of the strain. The species and locus of Cas protein in the four subtypes were predicted using CRISPRCasFinder, showing that they were identical in each subtype. Therefore, four strains were selected as representatives to display each CRISPR-Cas subtype (Figure 6a). The Cas3, Cas9, and Cas10 proteins were characteristic genes of type I, type II, and type III, respectively, which were labeled to distinguish different subtypes and were key proteins for the CRISPR-Cas system to target interference. In addition, Cas1 and Cas2 proteins were contained in each CRISPR-Cas system, which were responsible for the insertion of new spacer sequences as an important adaptive protein.

To analyze the functional coupling of the direct repeat (DR) sequence and the accompanying Cas protein, phylogenetic analysis was performed using the Cas1 and DR sequences. It showed that the same subtype concentrated on the same large branch, except for the IC subtype in the phylogenetic tree of Cas1 (Figure 6b). In addition, different DR sequences could be found by observing the CRISPR locus, with only individual base differences between them (Figure 6b). The WebLogo was used to visualize different repeats in the same CRISPR locus and two strains were selected as representatives for the display (Figure 6c). It showed that, although the repeat sequence was usually highly conserved throughout the locus, polymorphisms could be observed, notably for the terminal repeat. Specifically, sequence degeneracy was observed at the terminal repeat.

## 4. Discussion

*L. mucosae* is one of the potential probiotics colonized in the gut of humans and animals, and is also present in the human vagina. It has been reported that *L. mucosae* was not only used as a thickener and stabilizer in fermented food production [2] but also has health-associated benefits, such as improving immunity [36] and lowering cholesterol [10]. The currently published genomes of *L. mucosae* were limited and not sufficient for comparative genomic analysis. However, with the development of comparative genomics, the increase in genomic tools has provided strong support for subsequent diversity analysis. In the current work, the genetic diversity of 93 *L. mucosae* strains from different niches was analyzed with eight published *L. mucosae* genomes, and their functional diversity was explored.

The average genomic size of 93 *L. mucosae* was 2.11 Mb with a 48.07% average of the GC content, which was consistent with previous reports [12,13]. Unprecedented, the current work on the genome-wide 101 *L. mucosae* showed a trend of gradual closure of the genome [37]. This suggested that the genetic diversity or host adaptation of *L. mucosae* had reached its limitation, which supported the hypothesis that the relative size and contents of the pan-genome were potential indicators of the genetic plasticity and environmental adaptation potential of the species. In addition, by annotating the core genes, it revealed the functions and translations, defense mechanism, and general functional predictions.

A species was usually defined according to features encoded by the core genes, but did not adequately describe the genetic diversity specific to a particular species [38,39]. In 2012, Chan et al. [40] clearly distinguished 13 strains of Acinetobacter by ANI analysis (threshold 95%–96%) in combination with core gene phylogenetic trees, which had been shown to be suitable for different groups of bacteria. Therefore, the current study followed their method to identify *L. mucosae* with certain credibility. The ANI value of *L. mucosae* was between 0.955 and 0.999, which was consistent with a previous result [6]. Compared with other Lactobacilli, the ANI value span of *L. mucosae* was relatively large, indicating that the proportion of variable genes was large, and the diversity was rich. This may increase the bacterial selection advantage, such as adaptability to the different niches. By constructing a phylogenetic tree of 101 *L. mucosae* strains, it appeared to be some correlation between the phylogenetic relatedness and isolation origin of those strains but much less was anticipated. One possible reason for the phenomenon was that the number of non-human isolates was limited and not representative. Then, putting the gaze on the physical distance and age of the human source, it was found that the phylogenetic tree had a certain correlation with the physical distance, though without any obvious correlation with the age of the host. Specifically, with a smaller horizontal distance, the similarity among the strains was much higher. Under similar horizontal distances, coastal and inland areas were also factors influencing the genetic relationship of strains, which could not be ignored. Odamaki and colleagues [41] studied the isolates of *Bifidobacterium longum* subspecies and found no significant correlation with host age, which was similar to the current results. This could explain the wide adaptability of *L. mucosae* in different age groups. Unfortunately, considering that the strains isolated from middle-aged subjects accounted for a large proportion in this study, more strains from other age groups need to be added for further verification.

The ability to metabolize carbohydrates was an important indicator for the cultivation and selection of bacteria. Therefore, the ability of 93 strains to metabolize 24 sugars was determined. The utilization of 12 sugars (cellobiose, D-galactose, L-arabinose, maltose, D-mannose, melezitose, melibiose, raffinose, D-ribose, sucrose, trehalose, and D-xylose) was consistent with Bergey’s Manual of Systemic Bacteriology [42]. Contrary to the phenotypic results of esculin metabolism herein, it had been previously reported that eight strains of *L. mucosae* may utilize esculin [9]. The esculin operon consisted of two structural genes (*bglB* and *bglC*) and two regulatory genes (*bglR* and *bglS*) [43], which were not detected in *L. mucosae*. It was speculated that this difference may be caused by the insufficient strains in previous study, or the functional genes involved in esculin metabolism was lost during the host adaptation of those strains. Computer simulations of 25 GH families involved in carbohydrate metabolism revealed that GH13 and GH43 accounted for a relatively high proportion of the GHs, which were primarily involved in the degradation of starchy carbohydrates [44] and non-digestible dietary fibers [45,46]. This reflected to some extent the survival adaptability of *L. mucosae* in the intestine. In addition, the gene clusters associated with lactose [43], trehalose [47], D-fructose [48], and L-arabinose [49,50], emphasizing the importance of glycosyl hydrolase (*lacZ* and *treC*), isomerase (*araA* and *araD*), and kinases (*fruK*) in the corresponding operons. It was worth noting that *fruK*-encoded fructokinase was also important in the degradation of sucrose [51]. In addition, the strain FGSYC17L3, although containing the key gene, *treC*, was unable to utilize trehalose. It was probably due to the repression of transcription by trehalose operon repressor (*treR2*) located upstream, which mediated negative regulation. The PTS system or the ABC transporter was required in the metabolic system of fructose [48]. Although there was no complete PTS system in 13 strains of *L. mucosae*, the transport of fructose can be carried by ABC transporter permease. For arabinose, the transcriptional regulator was equivalent to the optical switch mechanism of the operon, and without it, the transcription of the arabinose operon cannot be promoted [52].

In recent years, EPS produced by lactic acid bacteria has attracted much attention. *L. mucosae* DPC6426 [2,3,4,10] and LM1 [6] were reported to generate EPS significantly, but the EPS gene cluster was not explored in depth. Genomic studies on lactic acid bacteria indicated that the biosynthetic pathway of EPS was controlled by several housekeeping genes and a series of EPS-related genes that were involved in regulating EPS production, chain-length, biosynthesis of repeating units, and aggregation and export of repeating unit [53,54,55,56]. In the current work, it was found that 82% of the strains lacked the chain length-determining protein, and it was speculated that these strains lost the ability to synthesize EPS. The glycosyltransferases involved in EPS synthesis were the GT2 family, which participated in the process of adding glycosyl groups to the growing EPS chain and directly determined that *L. mucosae* only produced a unique type of EPS [57]. It was worth noting that the third putative protein of the EPS gene cluster in some strains was missing. However, since the contribution of the hypothetical protein was not significant, it did not affect the synthesis of EPS [55]. Although discussion of EPS was still open, the fact that EPS clusters in *L. mucosae* has little change in the presence of particular genes should be highlighted.

*L. mucosae* AGR63 was reported to have the ability to produce only class III bacteriocin [58], and *L. mucosae* CRL573 was found to contain two potential enterostatin A operons (>10 kDa) [13]. Heretofore, little research has been done on the bacteriocin produced by *L. mucosae*. In the current work, 58.1% of *L. mucosae* contained the gene encoding bacteriocin, which was enterostatin A. This was consistent with the results reported previously. Enterolysin A is a heat-labile protein produced by *Enterococcus faecalis* LMG2333 and belongs to class III bacteriocin [59]. *EnlA* was identified as a key structural gene, which encoded enterolysin A [60], and all its upstream and downstream genes had no role in the synthesis of enterolysin A [58], revealing that the *enlA* gene was the unique gene in charge of producing enterolysin A. The evidence was provided for the structure of enterolysin A in *L. mucosae*. The presence of bacteriocin helped the strain to survive in a complex environment and provided it with a competitive advantage [61,62,63], which might be one of the advantages of *L. mucosae* as a potential probiotic in the intestine.

CRISPR loci presents in a large number of prokaryote genomes that provides acquired immunity against foreign genetic elements. Previously, little research had focused on CRISPR in *L. mucosae*, and only two strains were analyzed. In *L. mucosae* LM1, two CRISPR-Cas loci (type I and type II) were found [6] while only CRISPR elements were detected in *L. mucosae* CRL573 [13]. Therefore, the current work specifically analyzed the CRISPR-Cas system in all those 93 strains of *L. mucosae*, of which 44% contained the complete CRISPR-Cas system. The presence of the remaining incomplete loci may be due to genetic recombination, loss of activity to acquire other CRISPR loci, or incomplete assembly of the genomic sketches of these strains. The diversity of Cas protein was significant [64,65], but the CRISPR-Cas system could be easily classified (type I–III) by identifying the characteristic protein (Cas3, Cas9, and Cas10) in the gene composition [66]. The locus structure of the three subtypes (IE, IC, and IIA) of the CRISPR-Cas system was identical to the previously reported typical CRISPR-Cas system structure [67]. It was worth noting that type III was detected in nine strains of *L. mucosae*, which was firstly found, which will provide a new perspective for the future investigation of the CRISPR-Cas system in *L. mucosae*.

Previous studies have identified phylogeny of Cas1 as one of the key factors to subtype classification [68]. Additionally, the tree showed the aggregation of the Cas1 sequences was on the same big branch according to subtype IE, IIA, and IIIA, which was roughly the same as the phylogeny of the DR sequence, confirming the trend of co-evolution of components in the immune system [29]. The most frequent repeats were usually defined as typical repeats. Previous studies have defined two other types, namely “repeat variants” and “terminal repeats” [69]. The result was extremely important for proper annotation and orientation of the CRISPR locus, because the last repeat unit (which often contains degenerate terminal repeats) was frequently lost, or regularly repeated on the opposite DNA strand. In addition, transpositions have occurred at different positions of the CRISPR locus while transposases were associated with the frequent HGT in prokaryotes and had huge impacts on bacterial adaptation [70]. The existence of transposases showed the acquisition of a related gene structure to a certain extent, and it was an adaptive advantage for the survival of a complex niche, such as the human intestine.

## 5. Conclusions

A comparative genomics analysis for 101 strains of *L. mucosae* isolated from different niches was performed. The results showed that the genetic diversity of *L. mucosae* was related to the niches and physical distance but may be less affected by host age. Additionally, the genetic diversity of *L. mucosae* was reflected in carbohydrate metabolism and immune/competitive-related factors (EPS, enterolysin A and CRISPR-Cas). Among them, both the EPS operon and the IIIA-type CRISPR-Cas system were elaborated and discovered for the first time in *L. mucosae*. All the current results provide new information and a framework for the inheritance and diversity of *L. mucosae*.

## Figures and Tables

**Table 1:** Host information and general genome features of 101 strains of *L. mucosae.*

Strain	Region (City/Province)	Origin/Age	Gene Size	GC (%)	tRNA	Accession No.	Reference
			(Mb)				
DCC1HL5	Dali, Yunnan	Bovine Feces	1.96	48.17	57	SAMN13220078	This work
FAHBZ1M4	Bozhou, Anhui	Human feces, 24y	2.22	48.14	66	SAMN13220079	This work
FAHBZ5L1	Bozhou, Anhui	Human feces, 77y	2.05	48.17	62	SAMN13220080	This work
FAHBZ12M2	Bozhou, Anhui	Human feces, 6m	2.21	47.95	62	SAMN13220081	This work
FAHBZ16L4	Bozhou, Anhui	Human feces, 63y	2.02	47.85	52	SAMN13220082	This work
FAHBZ18M3	Bozhou, Anhui	Human feces, 26y	2.07	48.09	48	SAMN13220083	This work
FAHBZ20L1	Bozhou, Anhui	Human feces, 52y	2.00	47.84	54	SAMN13220084	This work
FAHBZ34M2	Bozhou, Anhui	Human feces, 51y	2.11	48.12	61	SAMN13220085	This work
FAHBZ49M2	Bozhou, Anhui	Human feces, 53y	2.01	48.21	53	SAMN13220086	This work
FAHBZ50M5	Bozhou, Anhui	Human feces, 14y	1.97	47.89	59	SAMN13220087	This work
FAHBZ53M2	Bozhou, Anhui	Human feces, 54y	2.01	48.08	47	SAMN13220088	This work
FGSYC1M1	Yongchang, Gansu	Human feces, 77y	2.13	48.07	50	SAMN13220089	This work
FGSYC15L3	Yongchang, Gansu	Human feces, 78y	2.07	47.84	52	SAMN13220090	This work
FGSYC16M1	Yongchang, Gansu	Human feces, 77y	2.07	47.68	55	SAMN13220091	This work
FGSYC17L3	Yongchang, Gansu	Human feces, 53y	2.11	47.73	48	SAMN13220092	This work
FGSYC19L1	Yongchang, Gansu	Human feces, 66y	2.17	47.98	54	SAMN13220093	This work
FGSYC23L4	Yongchang, Gansu	Human feces, 67y	2.14	47.98	54	SAMN13220094	This work
FGSYC27L1	Yongchang, Gansu	Human feces, 54y	2.07	48.23	50	SAMN13220095	This work
FGSYC42L4	Yongchang, Gansu	Human feces, 57y	2.09	47.96	48	SAMN13220096	This work
FGSYC43L5	Yongchang, Gansu	Human feces, 55y	2.33	48.08	58	SAMN13220097	This work
FGSYC90L5	Yongchang, Gansu	Human feces, 67y	2.22	48.1	52	SAMN13220098	This work
FHNXY1L8	Xiayi, Henan	Human feces, 65y	2.06	48.06	60	SAMN13220099	This work
FHNXY19M3	Xiayi, Henan	Human feces, 85y	2.14	47.98	49	SAMN13220100	This work
FHNXY29L2	Xiayi, Henan	Human feces, 66y	2.07	47.56	65	SAMN13220101	This work
FHNXY31L3	Xiayi, Henan	Human feces, 33y	2.06	48.08	44	SAMN13220102	This work
FHNXY47L1	Xiayi, Henan	Human feces, 86y	2.01	48.12	46	SAMN13220103	This work
FHNXY68L2	Xiayi, Henan	Dog feces	2.06	48.06	47	SAMN13220104	This work
FHNXY72L1	Xiayi, Henan	Dog feces	2.01	48.15	53	SAMN13220105	This work
FHuNHHMY89L8	Mayang, Hunan	Pig feces	2.25	47.84	53	SAMN13220106	This work
FJSCZ8L1	Changzhou, Jiangsu	Dog feces	1.99	48.23	42	SAMN13220107	This work
FJSNT61	Nantong, Jiangsu	Human feces, 82y	2.13	48.38	48	SAMN13220108	This work
FJSNT81	Nantong, Jiangsu	Human feces, 60y	2.20	48.1	47	SAMN13220109	This work
FJSNT91	Nantong, Jiangsu	Human feces, 79y	2.14	48.36	55	SAMN13220110	This work
FJSNT141	Nantong, Jiangsu	Human feces, 82y	2.05	48.26	61	SAMN13220111	This work
FJSNT152	Nantong, Jiangsu	Human feces, 95y	2.08	47.91	48	SAMN13220112	This work
FJSNT182	Nantong, Jiangsu	Human feces, 85y	2.27	47.65	56	SAMN13220113	This work
FJSNT192	Nantong, Jiangsu	Human feces, 84y	2.23	48.01	56	SAMN13220114	This work
FJSNT312	Nantong, Jiangsu	Human feces, 64y	2.04	48.04	59	SAMN13220115	This work
FJSNT321	Nantong, Jiangsu	Human feces, 63y	2.04	48.04	45	SAMN13220116	This work
FJSNT331	Nantong, Jiangsu	Human feces, 92y	2.18	48.1	66	SAMN13220117	This work
FJSWX5M5	Wuxi, Jiangsu	Human feces, 81y	2.12	48.22	50	SAMN13220118	This work
FJSWX8M3	Wuxi, Jiangsu	Human feces, 88y	2.08	48.07	50	SAMN13220119	This work
FJSWX13M2	Wuxi, Jiangsu	Human feces, 79y	2.17	48.21	56	SAMN13220120	This work
FJSWX15M1	Wuxi, Jiangsu	Human feces, 82y	2.08	47.81	59	SAMN13220121	This work
FJSWX18M1	Wuxi, Jiangsu	Human feces, 81y	2.2	48.15	56	SAMN13220122	This work
FJSWX19M2	Wuxi, Jiangsu	Human feces, 78y	2.12	47.83	62	SAMN13220123	This work
FJSWX20M1	Wuxi, Jiangsu	Human feces, 90y	2.16	47.84	57	SAMN13220124	This work
FJSWX21M1	Wuxi, Jiangsu	Human feces, 90y	2.17	48.08	49	SAMN13220125	This work
FJSWX27M1	Wuxi, Jiangsu	Human feces, 88y	2.05	47.68	60	SAMN13220126	This work
FJSWX28M2	Wuxi, Jiangsu	Human feces, 83y	2.05	47.84	62	SAMN13220127	This work
FJSWX34JL6	Wuxi, Jiangsu	Human feces, 3y	2.19	48.27	48	SAMN13220128	This work
FJSXYWG2L4	Xuyi, Jiangsu	Silage	1.97	48.33	41	SAMN13220129	This work
FJSXYWG6L3	Xuyi, Jiangsu	Cow feces	1.94	48.1	54	SAMN13220130	This work
FSH3M1	Shanghai	Human feces, 83y	1.99	48.37	41	SAMN13220131	This work
FSH12M2	Shanghai	Human feces, 84y	2.02	48.25	57	SAMN13220132	This work
FSH14M2	Shanghai	Human feces, 85y	2.10	48.17	54	SAMN13220133	This work
FSH18M1	Shanghai	Human feces, 85y	2.03	48.04	60	SAMN13220134	This work
FSH19M2	Shanghai	Human feces, 86y	2.04	48.23	59	SAMN13220135	This work
FSH21M1	Shanghai	Human feces, 87y	1.94	48.15	60	SAMN13220136	This work
FSH22M2	Shanghai	Human feces, 78y	2.02	48.16	60	SAMN13220137	This work
FSH26M1	Shanghai	Human feces, 71y	2.03	47.88	59	SAMN13220138	This work
FSH41M1	Shanghai	Human feces, 86y	2.09	48.24	59	SAMN13220139	This work
FSH46M2	Shanghai	Human feces, 84y	1.99	47.87	59	SAMN13220140	This work
FSH47M1	Shanghai	Human feces, 85y	2.08	47.88	58	SAMN13220141	This work
FYNLJ83L1	Lijiang, Yunnan	Pig feces	2.11	48.15	56	SAMN13220142	This work
FYNLJ87L2	Lijiang, Yunnan	Pig feces	2.10	47.97	49	SAMN13220143	This work
FZJTZ17M6	Taizhou, Zhejiang	Human feces, 67y	2.2	48.1	59	SAMN13220144	This work
FZJTZ18M3	Taizhou, Zhejiang	Human feces, 79y	1.86	48.03	64	SAMN13220145	This work
FZJTZ22M3	Taizhou, Zhejiang	Human feces, 55y	1.93	47.84	64	SAMN13220146	This work
FZJTZ23M9	Taizhou, Zhejiang	Human feces, 76y	2.45	47.71	46	SAMN13220147	This work
FZJTZ24M5	Taizhou, Zhejiang	Human feces, 62y	2.28	48.29	64	SAMN13220148	This work
FZJTZ26M3	Taizhou, Zhejiang	Human feces, 40y	1.97	48.59	44	SAMN13220149	This work
FZJTZ32M4	Taizhou, Zhejiang	Human feces, 58y	1.99	47.95	60	SAMN13220150	This work
FZJTZ34M1	Taizhou, Zhejiang	Human feces, 76y	2.20	48.16	50	SAMN13220151	This work
FZJTZ36M3	Taizhou, Zhejiang	Human feces, 80y	2.05	48.37	58	SAMN13220152	This work
FZJTZ60M1	Taizhou, Zhejiang	Human feces, 78y	2.25	48.3	53	SAMN13220153	This work
GD1M1	Lianzhou, Guangdong	Human feces, 75y	2.33	48.28	51	SAMN13220154	This work
GD2M2	Lianzhou, Guangdong	Human feces, 84y	2.33	48.15	58	SAMN13220155	This work
GD3M2	Lianzhou, Guangdong	Human feces, 76y	2.32	47.97	79	SAMN13220156	This work
GD4M1	Lianzhou, Guangdong	Human feces, 5y	2.13	48.06	38	SAMN13220157	This work
GD9M1	Lianzhou, Guangdong	Human feces, 76y	2.21	48.17	52	SAMN13220158	This work
GD14M3	Lianzhou, Guangdong	Human feces, 10y	2.36	48.18	55	SAMN13220159	This work
GD15M1	Lianzhou, Guangdong	Human feces, 10y	2.36	48.3	67	SAMN13220160	This work
GD16M9	Lianzhou, Guangdong	Human feces, 81y	2.36	48.23	56	SAMN13220161	This work
GD17M2	Lianzhou, Guangdong	Human feces, 8y	2.14	48.36	66	SAMN13220162	This work
GD22M1	Lianzhou, Guangdong	Human feces, 73y	2.15	48.23	57	SAMN13220163	This work
GD24M2	Lianzhou, Guangdong	Human feces, 58y	2.13	48.25	58	SAMN13220164	This work
GD64M2	Lianzhou, Guangdong	Human feces, 64y	2.05	48.19	55	SAMN13220165	This work
GD67M2	Lianzhou, Guangdong	Human feces, 9y	2.01	48.19	56	SAMN13220166	This work
GD68M4	Lianzhou, Guangdong	Human feces, 10y	2.00	48.31	53	SAMN13220167	This work
GD69M1	Lianzhou, Guangdong	Human feces, 63y	2.03	47.87	67	SAMN13220168	This work
M104R01L3	Dangxiong, Tibet	Yak milk	2.07	47.92	57	SAMN13220169	This work
VWX301M18	Wuxi, Jiangsu	Human vagina	2.01	47.84	61	SAMN13220170	This work
LM1	Brazil	Pig small intestine	2.43	46.13	91	SAMN02470226	[31]
DSM13345	Sweden	Pig small intestine	2.25	46.40	-	SAMN02369406	[32]
KHPC15	United States	Bovine rumen	1.88	46.70	46	SAMN05216545	[33]
KHPX11	United States	Bovine rumen	1.88	46.70	64	SAMN05216461	[33]
WCC8	United States	Bovine rumen	1.88	46.70	59	SAMN05216430	[33]
DPC6426	Ireland	Bovine rumen	2.80	47.00	89	SAMN03145820	[34]
AGR63	United States	Bovine rumen	1.94	47.00	94	SAMN02744693	[33]
L24-B	United States	Bovine rumen	2.00	46.90	88	SAMN10744154	[35]
